# Astrocytes in Neuropathic Pain: Mechanistic and Global Insights

**DOI:** 10.1155/prm/7987792

**Published:** 2026-09-18

**Authors:** Li Xue, Hong Yuan, Jiao Yang, Qiurui Yuan, Haixia Fan

**Affiliations:** ^1^ Department of Neurology, Norinco General Hospital, Xi’an, Shaanxi, China; ^2^ Department of Neurology, Dongguan Kanghua Hospital, Dongguan, Guangdong, China; ^3^ Department of Sleep Center, First Hospital of Shanxi Medical University, Taiyuan, Shanxi, China, sxmu.edu.cn

**Keywords:** astrocytes, bibliometric analysis, CiteSpace, neuropathic pain, VOSviewer

## Abstract

**Background:**

Neuropathic pain (NP) is a debilitating chronic pain condition caused by injury or disease of the somatosensory nervous system. Accumulating evidence indicates that astrocytes play a central role in neuroinflammatory regulation and synaptic remodeling, thereby critically influencing the initiation and persistence of NP. However, a comprehensive overview of research trends and knowledge structures in this field is still lacking.

**Methods:**

The analysis was conducted based on publications retrieved from the Web of Science Core Collection (WoSCC) and Scopus, covering the period from 2000 to 2025. Studies focusing on astrocytes and NP were systematically identified. Visualization and network analyses were performed using CiteSpace, VOSviewer, and the R package bibliometrix. Collaboration networks, cocitation patterns, keyword co‐occurrence, and thematic evolution were analyzed to delineate research hotspots, developmental trajectories, and scholarly contributions across countries, institutions, authors, and journals.

**Results:**

A total of 1828 publications were included, showing a 15% average annual growth in output, which accelerated post‐2010. The United States and China led in research and international collaboration, with studies concentrated in North American and East Asian institutions. Author productivity was uneven, with key researchers (Ji RR, Zhang Y, Watkins LR) contributing heavily to publications and citations. *Pain* and *Molecular Pain* were the core journals. Key themes included spinal astrocytic mechanisms, glial activation, and therapeutic modulation, with the focus evolving from injury models/markers to astrocytic activation and targeted pathways.

**Conclusion:**

Our analysis shows a substantial growth in astrocyte‐related NP research over the past 25 years, underscoring astrocytes’ key role in chronic pain pathophysiology. Current trends underscore the integration of mechanistic insights with translational relevance, thereby informing future therapeutic and mechanistic advancements in NP.

## 1. Introduction

Neuropathic pain (NP) is a complex, chronic pain condition that arises from damage to the nervous system, often resulting from injury, disease, or infection [1]. Epidemiological studies indicate that NP affects approximately 7%–10% of the general population, with a higher prevalence observed in individuals with conditions such as diabetes, multiple sclerosis, and postherpetic neuralgia. This type of pain can significantly impact quality of life, leading to increased healthcare utilization and associated economic burdens [2].

Astrocytes are increasingly recognized for their pivotal role in the pathophysiology of NP, influencing neuroinflammation, synaptic remodeling, and central sensitization [3]. Following nerve injury, activated astrocytes contribute to neuroinflammatory processes, leading to the production of proinflammatory factors that exacerbate pain signaling [4]. Their morphological and transcriptional changes, particularly in the dorsal horn, are associated with the amplification of NP signaling [5]. Additionally, astrocytes interact with microglia, further modulating neuroinflammation and central sensitization, which are key components in the development and maintenance of NP [6]. Targeting astrocytic pathways may offer therapeutic potential for alleviating NP by restoring balance in neuroinflammatory responses and pain signaling mechanisms [7]. Overall, astrocytes are emerging as increasingly critical players in the pathogenesis of NP.

Despite the growing body of literature on astrocytes in NP, a comprehensive overview of research trends and knowledge structures remains limited. Bibliometric analyses reveal an increasing interest in astrocytes across various neurological conditions; however, a focused analysis on NP is lacking [8]. Scientific mapping techniques have been employed in other fields to elucidate complex interactions, indicating a potential method to enhance the understanding of astrocytes in NP [9].

This study employs bibliometric analysis to systematically map the scientific landscape surrounding NP and astrocytes, revealing key research trends and emerging topics in the field. By identifying gaps and opportunities in current investigations, the findings will guide future research efforts aimed at developing targeted therapies that address astrocytic dysfunction in NP.

## 2. Methods

### 2.1. Search Strategy

**FIGURE 1:**
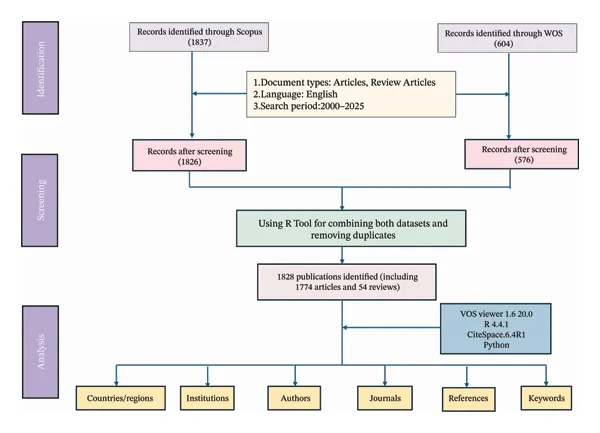
The flowchart for literature search, selection, and analysis.

Figure 1 illustrates the process of data retrieval and the criteria used for exclusion. Bibliometric data were collected by applying predefined search terms to the Web of Science Core Collection (WoSCC) and Scopus databases on November 2, 2025. The selection of search terms was based on Medical Subject Headings (MeSH) for term retrieval. The search was implemented in WoSCC using topic fields (TS), whereas identical keywords were searched within titles (TI), abstracts (AB), and author keywords (AK) for Scopus. Detailed information on the literature search strategies is provided in Supporting Table 3. By integrating multiple relevant keywords for the initial search, 604 records were retrieved from the WoSCC database, while 1837 records were obtained from Scopus.

After data screening, various nontarget document types were removed, encompassing proceedings papers, corrections, early access articles, news items, book chapters, retractions, reprints, biographical items, book reviews, meeting abstracts, editorial materials, and letters. The remaining dataset was limited to English‐language articles and reviews published between 2000 and 2025. After applying these criteria, 576 records from WoSCC and 1826 records from Scopus were retained (Figure 1).

Subsequently, using the bibliometrix package in R, the two datasets were combined, and duplicate records were subsequently screened based on matching titles, author lists, publication years, and DOIs. When DOIs were unavailable, duplicates were identified using a combination of article titles and first authors. All records flagged as potential duplicates through automated procedures were further examined manually to confirm their validity. This process ensured that publications indexed in both WoSCC and Scopus were counted only once. After this quality control step, a total of 1828 unique studies were included for subsequent analyses, comprising 1774 research articles and 54 review papers (Figure 1). These procedures were undertaken to minimize duplication bias and enhance the reliability of the dataset. Two reviewers independently conducted the screening of titles and abstracts, followed by full‐text evaluations according to predefined eligibility criteria. Discrepancies were resolved through consensus discussions. After the removal of duplicate records, 1828 unique studies remained for subsequent analysis.

### 2.2. Bibliometric and Scientometric Analysis

Bibliometric and scientometric investigations were conducted using multiple complementary software tools to improve the reliability of the analysis. CiteSpace (version 6.4R1, 64‐bit Advanced Edition) was utilized to construct visual knowledge maps illustrating authors, institutions, and keywords, employing a 1‐year time slicing strategy applied across the period from 2000 to 2025 [10]. In the analyses of authors and institutions, the top 25 most significant nodes in each time slice were retained. For keyword analysis, pathfinder pruning and network merging techniques were adopted to reduce network complexity and highlight the principal thematic structures. VOSviewer (version 1.6.20) was employed with the full counting method to visualize collaborative networks, including coauthorship patterns and interinstitutional collaborations [11]. Cluster analyses were performed using CiteSpace and VOSviewer to identify thematic and collaboration clusters. In CiteSpace, modularity Q and silhouette scores were used to evaluate cluster quality. Clusters with modularity *Q* > 0.3 and mean silhouette > 0.5 were considered statistically significant and well‐separated. VOSviewer automatically assigned cluster memberships based on association strength, with nodes colored according to their cluster identity. Additionally, the R package bibliometrix (https://www.bibliometrix.org) [12] was applied for historiographic analysis and for calculating key bibliometric indicators, such as the g‐index [13], h‐index [14], total citations (TC), and number of publications (NoP), thereby assessing research productivity and scholarly impact. Microsoft Excel 2021 was employed for initial data sorting and preprocessing.

## 3. Results

### 3.1. Annual Publication Trends

Overall, the publication output in astrocyte‐related NP research has exhibited exponential growth over the past 25 years, with a marked acceleration after 2010, reflecting the rapidly expanding recognition of astrocytes as critical players in pain pathophysiology.

**FIGURE 2:**
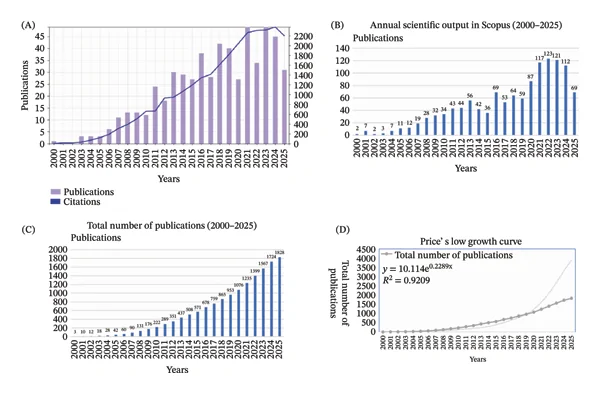
Publication trends in neuropathic pain research from 2000 to 2025. (A) Annual trends in the number of publications (bars) and citations (line) retrieved from the WoSCC database. (B) Annual trends in publications (bars) retrieved from the Scopus database. (C) Cumulative number of publications over time, illustrating the overall growth of the field. (D) Price’s law curve‐fitting analysis of cumulative publications, demonstrating the exponential growth pattern of astrocyte–neuropathic pain research.

The literature published between 2000 and 2025 revealed a marked upward trend in both publication output and citation frequency in the research field of astrocytes and NP. A total of 1828 records were retrieved from the WoSCC and Scopus databases, exhibiting an annual growth rate of 15.24%, which indicates a continuously expanding body of research (Supporting Table 1). As illustrated in Figure 2A, both annual publications and citations derived from the WoSCC saw steady growth in the early years, but there was a noticeable increase, with a sharp rise occurring after 2005. Figure 2B displays the trend from the Scopus database, which aligns with the trends shown in Figure 2A. Figure 2C illustrates the cumulative publication output over time, revealing a pronounced increasing trend, particularly over the past 2 decades. To quantitatively assess this expansion, a Price’s law–based growth curve fitting is shown in Figure 2D. The fitted exponential model, expressed as *y* = 10.114e^0.2289x^, achieves a high coefficient of determination (*R* ^2^ = 0.9209), indicating strong concordance between the model and empirical data. These results substantiate that the publication growth in this field follows an exponential pattern consistent with Price’s law, reflecting not only a substantial increase in research output but also the dynamic and rapidly advancing nature of the discipline.

### 3.2. Distributions of Countries/Regions

The United States and China emerged as the dominant contributors to this field, with distinct collaboration patterns and temporal trajectories that underscore the global distribution of research activity and the central role of transcontinental partnerships.

Figure 3A illustrates the global geographical collaboration network, and darker blue shading indicates higher research productivity. The United States and China act as core network nodes with extensive transcontinental cooperation. Figure 3B further visualizes collaborative relationships: The United States and China form two central clusters, connected to multiple countries (e.g., the United Kingdom, Germany, Australia), confirming their role as hubs of international collaboration. Figure 3C tracks long‐term publication trends for major contributors: U.S. annual research output rose steadily after 2000, whereas China’s publications grew slowly at first and increased rapidly after 2010; Italy, Japan, and South Korea achieved mild sustained growth and remain secondary to the United States and China by the 2020s. These temporal and cross‐national distribution trends are consistent with statistical data summarized in Supplementary Table 2. Figure 3D (Country collaboration and output distribution bar chart) quantifies country‐level output and collaboration metrics: The United States achieves the highest collaboration intensity, followed by China and South Korea; these three nations also lead overall research production. By comparison, Japan and Italy have medium collaboration and output levels, and smaller countries such as the Czech Republic and Finland contribute modestly to this field.

FIGURE 3Visualization and analysis of country/region and institutional involvement in neuropathic pain research. (A) Country/region collaboration map. Darker blue shading indicates higher research productivity, and the width of the links represents the strength of collaboration between two countries. (B) Country clustering analysis. Each node represents a country or region, with node size proportional to publication output. Edge thickness indicates the strength of coauthorship links between countries, while node colors denote collaboration clusters. (C) Top 5 countries/regions by publications. Lines illustrate the cumulative number of publications from 2000 to 2025. The *x* axis represents the year, and the *y* axis represents the cumulative document count. (D) Leading countries by the number of studies and collaboration type. Teal segments represent single‐country publications (SCP), while red segments represent multiple‐country publications (MCPs). (E) Institutional collaboration network. Node size corresponds to the extent of collaboration activity, and distinct colors denote clusters, generally reflecting regional or institutional groupings. Line thickness indicates the intensity of cooperation. (F) Institutional publication trends. The *x* axis represents years, and the *y* axis represents publication counts.

**Figure:**
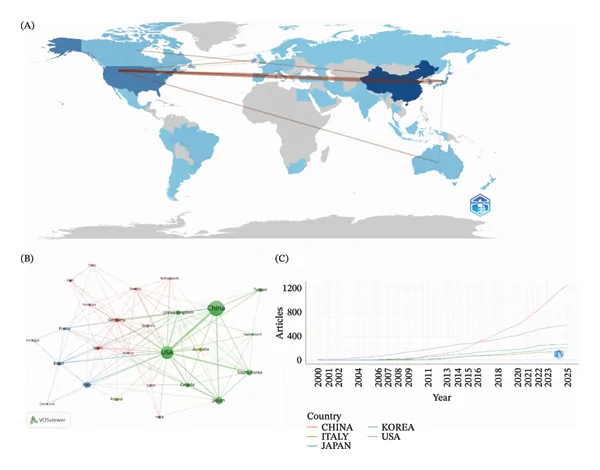

**Figure:**
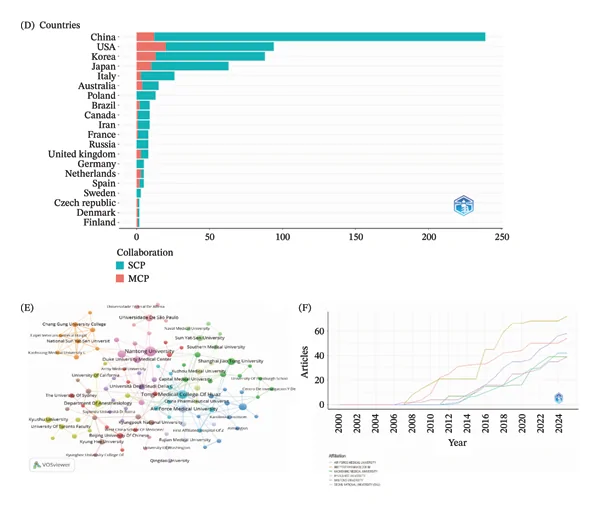

### 3.3. Characteristics of the Institutional Collaboration Network

Institutional collaboration exhibits a multicluster structure characterized by geographically concentrated domestic networks and selective international bridging, with a few high‐output institutions serving as critical nodes for cross‐regional knowledge exchange.

Figure 3E visualizes the collaborative relationships among institutions in the field, where node size corresponds to research output, and edge connections represent collaborative ties. The network exhibits a multicluster structure with geographically and thematically linked institutional groups: A prominent cluster centered on Chinese institutions (e.g., Nantong University, Sun Yat‐Sen University, Shanghai Jiao Tong University) forms a dense collaborative subnetwork, reflecting intensive domestic partnerships in the field. North American institutions (e.g., University of California, Duke University Medical Center, University of Washington) constitute another key cluster, connected both internally and to select international partners. Scattered nodes (e.g., University of São Paulo [Brazil], Karolinska Institutet [Sweden], University of Sydney [Australia]) represent institutions with relatively independent collaboration patterns, linking to core clusters via limited cross‐regional ties. Notably, institutions with larger nodes (e.g., University of California, Sun Yat‐Sen University) act as “bridging nodes,” connecting multiple subclusters and facilitating knowledge exchange across geographic regions. Figure 3F illustrates the cumulative publication output of key institutions from 2000 to 2025. Among the profiled institutions, Instytut Farmakologii (yellow curve) exhibited the most significant growth, with a sharp increase in annual publications post‐2016. Air Force Medical University, Instytut Farmakologii, Kaohsiung Medical University, and Nantong also demonstrated accelerated growth after 2010, while Seoul National University (SNU) (purple curve) maintained steady, moderate expansion throughout the period.

### 3.4. Author Collaboration and Publication Patterns

Author productivity follows a highly skewed distribution consistent with Lotka’s law, with a small group of core researchers driving both publication output and citation impact, while collaborative networks reveal distinct regional and thematic subgroups.

Table 1 quantifies the influence of leading authors in NP research (2000–2025), featuring metrics including h‐index, g‐index, and TC. The h‐index (a widely adopted bibliometric indicator) reflects consistent research productivity by measuring how many of an author’s papers have been cited at least h times [14]; the g‐index, by contrast, prioritizes highly cited works to highlight impact in key research domains [13]. For example, JI RR (Rank 1) holds an h‐index of 33 and a g‐index of 37, alongside a TC of 11,671—these metrics underscore his sustained, high‐impact contributions to the field since 2003. ZHANG Y (Rank 2) demonstrates a stronger concentration of influential works: With an h‐index of 28 and a g‐index of 50 (the highest g‐index among top authors), his output reflects a broader citation reach despite a later research start in 2007. Notably, WATKINS LR (Rank 5) achieves a high TC of 6052 with an h‐index of 22, indicating that his work has garnered substantial attention in core research areas.

**TABLE 1:** Top 10 authors on astrocytes in neuropathic pain research (2000–2025).

Rank	Author	h_index	g_index	m_index	TC	NoP	PY_start	TP	FP
1	JI RR	33	37	1.435	11,671	37	2003	37	8.99
2	ZHANG Y	28	50	1.474	2528	57	2007	57	8.28
3	WANG Y	24	37	1.412	1512	54	2009	54	8.28
4	MIKA J	22	27	1.222	1974	27	2008	27	4.65
5	WATKINS LR	22	26	0.957	6052	26	2003	26	4.20
6	ZHANG J	21	34	1.050	1570	34	2006	34	5.66
7	GAO Y	20	32	1.111	3577	32	2008	32	5.83
8	LI Y	20	33	1.176	1169	46	2009	46	5.52
9	WANG W	19	26	1.056	919	26	2008	26	3.35
10	GHELARDINI C	18	24	1.385	1091	24	2013	24	3.25

*Note:* PY_start: year of first publication.

Abbreviations: FP, fractionalized publications; NoP, number of publications; TC, total citations; TP, total publications.

**FIGURE 4:**
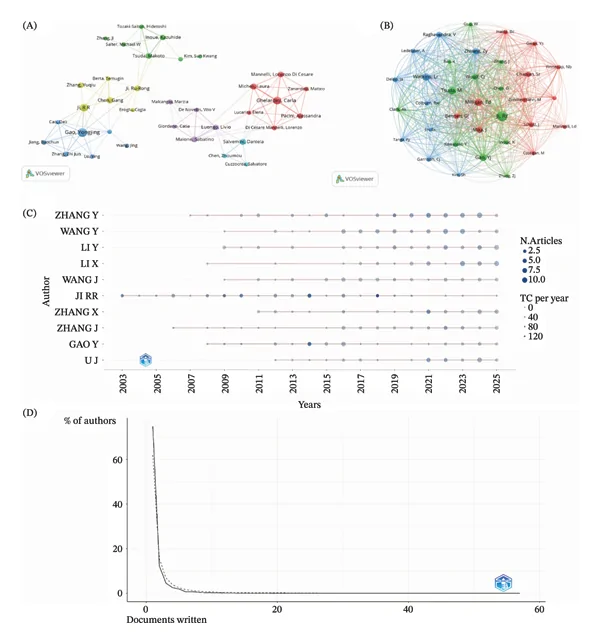
Visualization and analysis of author networks and cocited authors in neuropathic pain. Coauthorship and cocited authorship networks on astrocytes in the neuropathic field (2000–2025). (A) Coauthorship network: Nodes represent authors, sized by publication count. Colors indicate collaboration clusters. (B) Cocited authorship network: Nodes, connected by collaborative publications, have edge thickness proportional to joint works. Clusters, marked by colors, reveal research groups. (C) Annual publication trends of the top 10 authors. (D) Lotka’s law fitting curve: highlights the distribution of productivity and collaboration patterns among authors.

Author collaboration networks (Figure 4A,B) reveal distinct collaborative clusters within the research community. In Figure 4A, multiple small‐to‐medium collaborative subgroups are observed (e.g., the cluster centered on Gaol, Yongjing; the European cluster led by Ghelardini, Carla), indicating relatively concentrated regional/team‐based collaborations. Figure 4B further expands the scope, showing a denser and more interconnected collaboration network: Core authors (e.g., Ji Rr, Watkins Lr, Mannelli Ld) act as bridges across multiple clusters, whereas subgroups (e.g., the Gao, Y J cluster, Zimmermann, M cluster) reflect specialized research teams with close internal collaborations. The annual publication and temporal citation trends of high‐output authors (Figure 4C) show that core authors (e.g., ZHANG Y‐, WANG Y‐) maintained stable annual publication output from 2007 to 2023. For JI RR, two notable citation peaks (around 2015 and 2019) correspond to high‐impact works in these periods; authors such as ZHANG X‐ showed a gradual increase in annual output in recent years (post‐2019), indicating growing research activity. The author productivity distribution (Figure 4D) follows a typical Pareto principle: The vast majority of authors (over 90%) published ≤ 5 documents, whereas only a small proportion of core authors contributed ≥ 10 documents. This skewed distribution (a rapidly declining curve in the low‐document range) reflects the “long tail” feature of author productivity in this field—research output is concentrated among a small number of high‐productivity scholars.

### 3.5. Journals and Cojournals

Research dissemination in this field is concentrated in a limited set of core journals, with Pain and Molecular Pain serving as the primary publication platforms, and journal cocitation patterns revealing three main thematic clusters spanning basic neuroscience, molecular research, and clinical translation.

**FIGURE 5:**
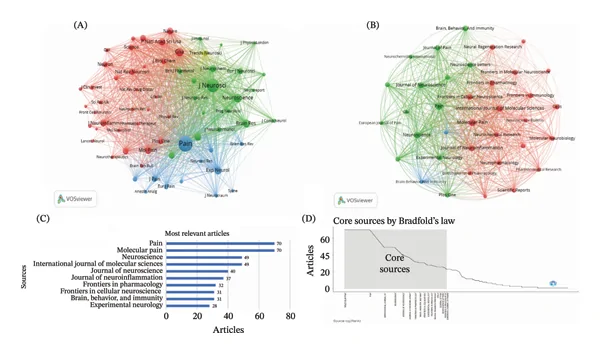
Visualization of journal association networks in neuropathic pain research. (A) Journal citation network: Nodes represent journals, connected by citations. Colors (green, blue, red) indicate thematic clusters of frequent intercitation. (B) Journal cocitation network: Nodes, sized by cocitation frequency, are linked by cocitation ties. Colors denote clusters of commonly cocited journals. (C) The *x* axis shows article numbers, while the *y* axis lists the journals. (D) Bradford’s law analysis: identifies core journals based on publication distribution.

The journal collaboration network (Figure 5A,B) reflects the pattern of cross‐journal academic exchange in this field. In Figure 5A, journals are clustered into three main groups (red, green, and blue), with Pain as the core node (blue cluster) connecting multiple journals (e.g., *Eur J Pain*, *Exp Neurol*), indicating its central position in interdisciplinary exchanges. Table 2 further substantiates their scholarly influence. The leading status of these journals is demonstrated not only by their substantial publication output but also by their strong academic visibility and reputational standing. The majority are classified as Q1 journals in the JCR, underscoring their significant impact and broad recognition within the academic community. Figure 5B further expands the network: The green cluster (centered on the Journal of *Neuroscience* and *Pain*) dominates basic neuroscience and pain studies, with dense internal links. The red cluster (e.g., *Scientific Reports*, *Cells*) focuses on molecular/translational research, closely connecting with the green cluster. *Pain* acts as a core node bridging multiple subfields, reflecting the field’s integrated academic exchange.

**TABLE 2:** Top 15 influential journals on astrocytes in neuropathic pain research (2000–2025).

Rank	Source	h_index	g_index	m_index	TC	NoP	PY_start	IF	JCR
1	Pain	44	70	1.76	6154	70	2001	5.5	Q1
2	Journal of Neuroscience	32	40	1.391	7089	40	2003	4	Q1
3	Neuroscience	30	49	1.154	3312	49	2000	2.8	Q1
4	Molecular Pain	28	43	1.556	2081	70	2008	2.8	Q1
5	Journal of Neuroinflammation	26	37	1.625	2510	37	2010	10	Q1
6	Experimental Neurology	24	28	1.143	2539	28	2005	4.2	Q2
7	Brain Behavior and Immunity	22	31	1.158	1968	31	2007	7.6	Q1
8	International Journal of Molecular Sciences	22	35	2	1347	49	2015	4.9	Q1
9	Journal of Pain	19	26	0.792	1647	26	2002	4	Q1
10	Frontiers in Cellular Neuroscience	18	31	1.385	1707	31	2013	4	Q2
11	Journal of Neurochemistry	18	22	0.783	1690	22	2003	4	Q2
12	Frontiers on Immunology	17	28	1.889	831	28	2017	5.9	Q1
13	European Journal of Pharmacology	16	23	0.889	1269	23	2008	4.7	Q2
14	Frontiers in Pharmacology	16	28	1.778	795	32	2017	4.8	Q1
15	PLOS One	16	20	0.941	1135	20	2009	2.6	Q1

Abbreviations: IF, impact factor; JCR, Journal Citation Reports.

The distribution of high‐output journals (Figure 5C) shows that *Molecular Pain* and *Pain* are the most relevant sources (with 70 published documents each), followed by *International Journal of Molecular Sciences* and *Neuroscience* (with 49 documents). This indicates that top‐tier journals in pain research and molecular neuroscience serve as the primary publication platforms for this field. Based on Bradford’s law (Figure 5D), the journal distribution follows a segmented pattern: A small number of “Core Sources” (the shaded area) publish the majority of documents in this field, while the number of journals decreases sharply as publication volume declines. This result confirms that research output in this field is concentrated in a limited set of core journals, which play a leading role in academic dissemination.

### 3.6. Citation Network and Thematic Evolution of NP Research

Co‐citation and cluster analyses reveal a clear temporal evolution from foundational anatomical and molecular studies (2000–2010) toward mechanistic signaling research (2011–2020) and recent translational frontiers (2021–2025), with key scholars such as Ji RR maintaining sustained influence across multiple thematic stages.

The research on astrocytes in NP is influenced by prominent authors, high‐impact journals, and highly cited studies. As shown in Supporting Table 1, the analysis included 1828 publications with 30,801 TC (2000–2025), resulting in a mean age of 7.23 years and an average of 55.86 citations per article, indicating strong scholarly influence and sustained academic relevance.

The citation collaboration network (Figure 6A) illustrates the knowledge connection pattern of NP studies from 2000 to 2025. The ten most highly cited publications are summarized in Table 3. Core references (e.g., Ji RR (2019) [15], Donnelly CR (2020) [16], Finnerup NB (2021) [17]) are positioned at the center of the network, characterized by larger nodes and dense links, which indicate their high citation frequency and central status in academic discourse. Notably, Ji RR (with multiple nodes in 2013/2016/2018/2019) forms a key connection hub, reflecting the author’s sustained influence across different stages of research.

FIGURE 6Network visualization of references in neuropathic pain. (A) Reference co‐occurrence network based on CiteSpace, with nodes representing references, node size indicating frequency, and connections illustrating co‐occurrence relationships. (B) Clustered co‐citation network: Nodes, sized by citation frequency, are grouped by modularity with keyword labels for major clusters. Edge thickness reflects cocitation strength, while colors highlight thematic diversity. (C) Timeline clustering of publications. (D) Top 25 references with the strongest citation bursts in the research. The blue lines indicate the time intervals, while red lines mark the periods of burst activity for each reference, showing the initial and final years of the citation burst duration.

**Figure:**
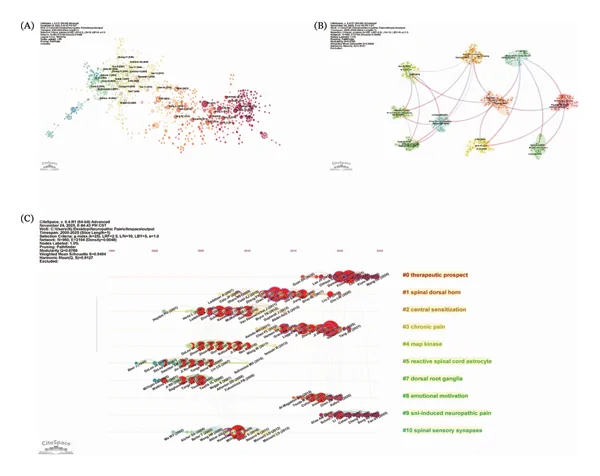

**Figure:**
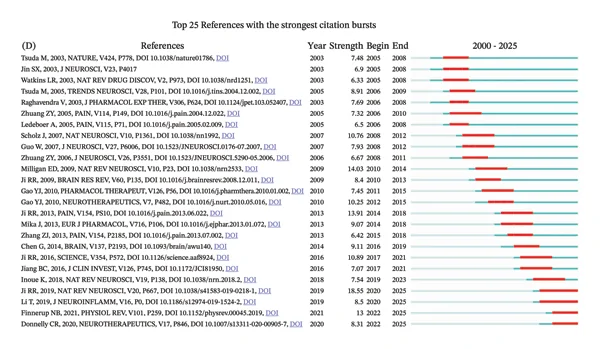

**TABLE 3:** Top 10 most cited publications based on bibliometric analysis (2000–2025).

Rank	First author	Year	Journal	Paper	DOI	Total citations	TC per year	Normalized TC
1	SOFRONÏEW MV	2010	ACTA NEUROPATHOL	Astrocytes: Biology and pathology	10.1007/s00401‐009‐0619‐8	4197	262.31	19.35
2	RANSOHOFF RM	2009	ANNU REV IMMUNOL	Microglial physiology: Unique stimuli, specialized responses	10.1146/annurev.immunol.021908.132528	1547	91.00	8.60
3	SCHOLZ J	2007	NAT NEUROSCI	The neuropathic pain triad: Neurons, immune cells and glia	10.1038/nn1992	1534	80.74	8.15
4	BURNSTOCK G	2007	PHYSIOL REV	Physiology and pathophysiology of purinergic neurotransmission	10.1152/physrev.00043.2006	1387	73.00	7.37
5	MILLIGAN ED	2009	NAT REV NEUROSCI	Pathological and protective roles of glia in chronic pain	10.1038/nrn2533	1231	72.41	6.84
6	JI RR	2018	ANESTHESIOLOGY	Neuroinflammation and central sensitization in chronic and widespread pain	10.1097/ALN.0000000000002130	1103	137.88	19.92
7	JI RR	2014	NAT REV DRUG DISCOV	Emerging targets in neuroinflammation‐driven chronic pain	10.1038/nrd4334	874	72.83	10.59
8	JI RR	2009	BRAIN RES REV	MAP kinase and pain	10.1016/j.brainresrev.2008.12.011	845	49.71	4.70
9	JIN S	2003	J NEUROSCI	p38 mitogen‐activated protein kinase is activated after a spinal nerve ligation in spinal cord microglia and dorsal root ganglion neurons and contributes to the generation of neuropathic pain	10.1523/jneurosci.23‐10‐04017.2003	794	34.52	1.51
10	GRACE PM	2014	NAT REV IMMUNOL	Pathological pain and the neuroimmune interface	10.1038/nri3621	751	62.58	9.10

Abbreviation: DOI, digital object identifier.

The cluster dependency diagram (Figure 6B) identifies 10 distinct thematic clusters: #0 therapeutic prospect; #1 spinal dorsal horn and #7 dorsal root ganglia (anatomical basis of pain signal transmission); #2 central sensitization and #3 chronic pain (pathophysiological mechanisms of pain persistence); #4 mitogen‐activated protein kinase (MAPK) (molecular signaling pathways); #5 reactive spinal cord astrocyte (glial cell role in pathogenesis); #8 emotional motivation (pain–psychological links); #9 spared nerve injury (SNI)–induced NP (experimental models); and #10 spinal sensory synapses (synaptic transmission). Cross‐cluster links (e.g., central sensitization–spinal dorsal horn) reflect the interdisciplinary integration of mechanisms and anatomical targets.

The cluster timeline (Figure 6C) illustrates the temporal thematic evolution: 2000–2010 (early stage), clusters (#7, #10) established anatomical/molecular foundations; 2010–2020 (midstage) saw the rise of functional mechanism clusters (#2, #4); and 2020–2025 (recent stage) shifted to translational research and model optimization (#0, #9). Ji RR contributed to multiple clusters over time, underscoring continuous contributions to key fronts.

Figure 6D ranks the top 25 references by citation burst strength. Milligan and Watkins [18] demonstrated the strongest burst (strength = 14.03, 2010–2014), laying a foundation for early 2010s research. High‐impact references also include Ji et al. [15], (strength = 18.55, 2020–2025) and Scholz and Woolf [19] (strength = 10.76, 2008–2012). Early bursts (e.g., Tsuda et al. [20] and Zhuang et al. [21]) focused on basic mechanisms, while mid‐to‐late bursts (e.g., Ji et al. [22] and Finnerup et al. [17]) leaned toward translation and clinical research. These bursts trace the field’s evolution from basic mechanisms to translational applications, with core scholars such as Ji RR driving high‐impact fronts.

### 3.7. Keyword Analysis

Keyword analysis identifies core themes and evolutionary trends in NP research (2000–2025) via VOSviewer and CiteSpace. A total of 11,601 Keywords Plus and 3576 author keywords were analyzed, comprehensively characterizing research foci (Supporting Table 1).

Keyword cooccurrence and burst analyses identify a shift from early focus on injury models and glial markers toward emerging themes including oxidative stress, synaptic plasticity, sex differences, and spinal cord injury (SCI), reflecting the field’s maturation toward circuit‐level and personalized mechanistic investigations.

**FIGURE 7:**
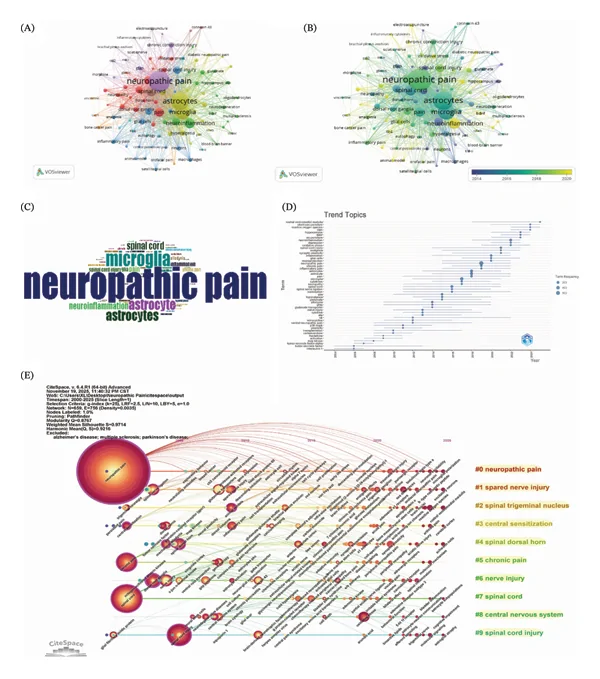
Visualization of keywords in neuropathic pain research. (A) Co‐occurrence clustering of keywords and (B) average normalized citations in author keyword analysis. Each circle and its associated label represent a keyword unit, with different colors indicating distinct clusters. (C) Author keyword word cloud: The size of each word reflects its frequency. (D) Keyword timeline: Nodes, sized by frequency within each cluster, illustrate the temporal evolution of research topics. (E) Cocitation timeline: Clusters are shown as horizontal lines, with node size reflecting cocitation frequency, links indicating relationships, and node positions marking the first cocitation year.

As shown in Figure 7A,C, “neuropathic pain” (term frequency = 1393, total link strength = 1427) is the dominant central theme, closely associated with “astrocytes” (frequency = 1718, link strength = 1174) and “microglia” (frequency = 906, link strength = 952). Other high‐frequency terms include “spinal cord” (490) and “neuroinflammation” (426). Figure 7B reveals dynamic shifts: Pre‐2016 focus on model‐based terms (e.g., “chronic constriction injury [CCI]”) shifted post‐2018 to “oxidative stress” and “diabetic neuropathic pain,” expanding into comorbid and mechanistic dimensions. Figure 7D (trend topic plot, 2004–2024) illustrates temporal thematic evolution: 2004–2012 (early phase) centered on basic inflammatory mediators (e.g., tumor necrosis factor‐alpha, interleukin‐1) with low frequencies (< 200); 2012–2018 (mid phase) saw rapid growth of core themes (e.g., NP, astrocytes, frequency > 400); 2018–2024 (late phase) witnessed surging frontier topics (e.g., electroacupuncture, reactive oxygen species, frequency > 600), reflecting an interest in nonpharmacological therapies and novel mechanisms.

**FIGURE 8:**
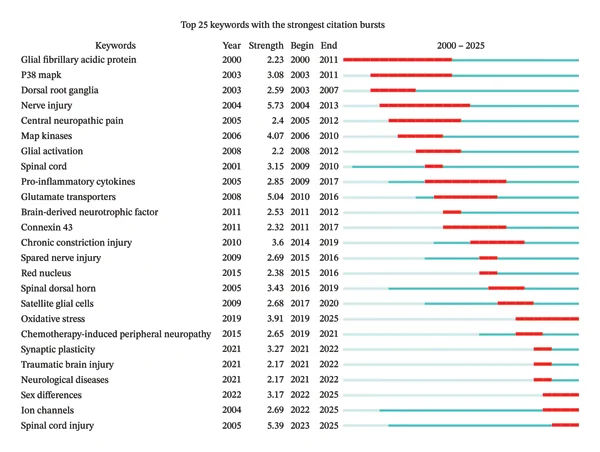
Top 25 keywords with the strongest citation bursts in neuropathic pain research. The burst timeline (right‐hand side) displays the periods during which these bursts occurred, with red segments indicating the time intervals when each reference received heightened attention. The burst detection process highlights key works that had a significant impact in a short time, suggesting pivotal contributions to the evolving research landscape in neuropathic pain.

Using CiteSpace for keyword burst analysis (Figure 8), we can delineate temporal shifts in research hotspots and cutting‐edge directions in NP studies (2000–2025), while identifying emerging trends. Based on burst timing and intensity, the top 25 keywords with the strongest citation bursts can be categorized into three phases, reflecting distinct stages of academic evolution. In the early phase (2000–2010), research focused on foundational mechanisms and anatomical correlates of pain. Keywords like glial fibrillary acidic protein (GFAP) (burst strength = 2.23, 2000–2011) and dorsal root ganglia (burst strength = 2.59, 2003–2007) exhibited significant bursts, highlighting early attention to glial cell markers and peripheral pain‐signaling structures. Nerve injury (burst strength = 5.73, 2004–2013) emerged as a core theme, underscoring the field’s initial focus on injury‐induced pain models and basic pathophysiology. The middle phase (2011–2020) saw a shift toward mechanistic and therapeutic exploration. Glutamate transporters (burst strength = 5.04, 2010–2016) and CCI (burst strength = 3.6, 2014–2019) gained traction, reflecting deepened interest in synaptic neurotransmission and preclinical pain models. Satellite glial cells (burst strength = 2.68, 2017–2020) and oxidative stress (burst strength = 3.91, 2019–2025) also exhibited late‐phase bursts during this period, signaling a growing focus on glial‐mediated inflammation and redox‐related pain mechanisms. By the late phase (2021–2025), the field shifted toward translational and specialized subdomains. Synaptic plasticity (burst strength = 3.27, 2021–2022) and sex differences (burst strength = 3.17, 2022–2025) emerged as new areas of interest, emphasizing the significance of circuit‐level pain adaptation and personalized pain responses. Notably, SCI (burst strength = 5.39, 2023–2025) exhibited the strongest late‐phase burst, reflecting a renewed focus on comorbid pain in neurological trauma—aligning with clinical demands for integrated injury‐pain management.

## 4. Discussion

This bibliometric study provides, for the first time, a systematic mapping of the scientific landscape linking astrocytes to NP over the past 25 years. Unlike traditional narrative reviews, our analysis quantifies publication growth, collaborative networks, thematic evolution, and emerging frontiers based on 1828 unique publications. The results reveal an exponential growth trajectory (*R* ^2^ = 0.9209; Figure 2D), accelerating after 2010, which reflects the increasing recognition of astrocytes as key players in NP pathophysiology. Below, we interpret our major bibliometric findings—core research themes, temporal shifts, etiology‐specific patterns, and citation bursts—and link them to the underlying pathological mechanisms and therapeutic targets.

### 4.1. Core Research Themes: From Reactive Gliosis to Molecular Pathways

Our cluster analysis identified three dominant and enduring research themes (Figure 6B,C). The largest cluster (#5, “reactive spinal cord astrocyte”) centers on GFAP upregulation and astrocyte morphological changes following nerve injury [23, 24]. This theme, which accounted for the highest number of publications in the early 2000s, directly aligns with foundational studies showing that spinal nerve ligation or CCI induces GFAP expression in the dorsal horn [25]. However, our cocitation timeline (Figure 6C) shows that this descriptive morphological focus gradually integrated with mechanistic clusters after 2010.

The second major theme (#2 “central sensitization” and #4 “map kinase”) reflects a shift toward molecular signaling. Keywords such as “p38 MAPK” and “neuroinflammation” surged between 2010 and 2020 (Figures 7 and 8). Notably, the burst detection identified “glutamate transporter” (strength = 5.04, 2010–2016) and “oxidative stress” (strength = 3.91, 2019–2025) as high‐impact terms, indicating that astrocytic regulation of glutamate homeostasis and redox balance became recognized as central‐to‐central sensitization [26, 27]. The convergence of these themes in our network demonstrates that astrocytes are no longer viewed as passive scar‐forming cells but as active modulators of synaptic transmission and neuroinflammation.

The third theme, which emerged after 2015 (#0 “therapeutic prospect” and #1 “spinal dorsal horn”), emphasizes translational attempts. High‐frequency keywords such as “electroacupuncture” and “reactive oxygen species” (Figure 7D) together with burst terms “sex differences” (2022–2025) and “spinal cord injury” (2023–2025) (Figure 8) indicate that recent research is moving toward mechanism‐based interventions and personalized pain management.

### 4.2. Temporal Evolution: From Injury Models to Circuit‐Level Mechanisms

The timeline analysis (Figures 6C and 7E) reveals a clear chronological progression. Between 2000 and 2010, publications were dominated by anatomical and model‐based terms: “Dorsal root ganglia,” “nerve injury,” and “chronic constriction injury” exhibited the strongest bursts during this period (Figure 8). Research focused on establishing that peripheral nerve damage leads to spinal astrocyte activation. From 2011 to 2020, the field transitioned to dissecting intracellular signaling cascades. The burst of “satellite glial cells” (2017–2020) and “oxidative stress” (2019–2025) marks a broadening of glial targets beyond spinal cord astrocytes to dorsal root ganglia glia and a deeper exploration of redox‐dependent mechanisms. Since 2021, the most striking shifts are “synaptic plasticity” (2021–2022) and “sex differences” (2022–2025). This temporal pattern strongly suggests that the field has matured from descriptive phenomenology to circuit level, sex aware, and translational science.

### 4.3. Etiology‐Dependent Astrocytic Mechanisms: A Missing Link Revealed by Bibliometric Subanalysis

Posttraumatic nerve injury and SCI: They represent the largest proportion. Traumatic nerve injury induces rapid astrocytic reactivity in the spinal dorsal horn, with CXCL13/CXCR5 signaling and MAP kinase pathways (cluster #4, Figure 6B) serving as central mechanisms [28, 29]. The recent burst of “spinal cord injury” (2023–2025, Figure 8) is interpreted in the context of comorbid pain management in trauma patients, highlighting the clinical urgency of this research direction.

Diabetic neuropathy: Diabetic neuropathy shows a different signature. Astrocytic activation in diabetic neuropathy is characterized by metabolic stress‐induced GFAP upregulation, advanced glycation end‐product (AGE) signaling, and impaired glutamate transporter function (specifically excitatory amino acid transporter 2 [EAAT2]/glutamate transporter 1 [GLT‐1] downregulation) [30, 31]. We link these findings to the keyword burst trends (Figure 8) showing “diabetic neuropathic pain” as an emerging theme post‐2018, and this suggests that in diabetic NP, astrocytic dysfunction centers on impaired glutamate uptake and metabolic dysregulation rather than on pure proliferative gliosis.

Chemotherapy‐induced peripheral neuropathy (CIPN): It is the fastest‐growing category after 2018 (Figure 7B, trend topic plot). Astrocytes in CIPN models exhibit distinct phenotypic changes, including mitochondrial dysfunction, oxidative stress (reflected in the “oxidative stress” keyword burst, 2019–2025), and altered cytokine profiles (tumor necrosis factor‐α [TNF‐α], interleukin‐1β [IL‐1β]) [32, 33]. We connect the temporal surge in CIPN‐related publications post‐2015 to increased clinical awareness and the development of astrocyte‐targeted neuroprotective strategies.

Viral and infectious neuropathies: We address the emerging bibliometric signals for postherpetic neuralgia and HIV‐associated neuropathy, noting that while these subfields remain relatively small, they show increasing astrocytic involvement in neuroinflammatory maintenance of pain [34, 35], as reflected in the cocitation network (Figure 6A).

### 4.4. Emerging Frontiers and Future Directions

• Synaptic plasticity (burst 2021–2022) is rapidly gaining attention. Astrocytes regulate synaptic strength through release of gliotransmitters (e.g., ATP, D‐serine) and by modulating glutamate spillover. The recent burst of “N‐myc downstream regulated gene 2 (NDRG2)/GLT 1 pathway” exemplifies this direction [36]. Future studies should combine single synapse imaging with cell‐type‐specific manipulations in chronic pain models.
• Sex differences (burst 2022–2025) are a late‐stage emerging theme. Our data show that only a handful of papers currently include sex as a biological variable, but the citation burst strength (3.17) indicates rapid uptake. Given that female rodents exhibit different astrocytic responses to nerve injury [37, 38] and that clinical NP prevalence is higher in women [39], this area demands dedicated mechanistic investigation.
• SCI‐induced NP (burst strength = 5.39, 2023–2025) has re‐emerged as a strong frontier. Unlike peripheral nerve injury, SCI involves supraspinal astrocyte reactivity and a prolonged inflammatory milieu. The burst aligns with a renewed clinical need for treatments targeting below‐level NP. Our cocitation network (Figure 6A) shows that recent high‐impact reviews [17] are heavily cited together with astrocyte‐specific papers, indicating an integrative trend.
• Nonpharmacological interventions such as acupuncture (rising in Figure 7D) and tuina [36] have appeared as high‐frequency keywords. While these are not drug‐based, their mechanistic link to astrocytic glutamate transporters (e.g., NDRG2/GLT 1) provides a biological rationale. Future bibliometric updates will likely show further growth in this area.

### 4.5. Limitations

Despite offering a thorough examination of publication patterns and collaborative structures within the field, this study has several acknowledged limitations. First, the analysis is largely based on records obtained from WoSCC and Scopus, which may not fully capture all pertinent literature in this domain. Second, the scope of this study was restricted to English‐language literature, potentially leading to the underrepresentation of significant research contributions published in other languages and regions. Additionally, a noticeable geographical imbalance was observed in the distribution of research output, with the United States and China dominating the field. This imbalance suggests that the findings may not fully reflect the global landscape of research activity and investment. To address these limitations, future studies should broaden the range of data sources by incorporating additional bibliographic databases and including multilingual and regionally diverse literature, thereby providing a more comprehensive and balanced assessment of global scholarly influence in this field.

## 5. Conclusion

Overall, this study demonstrates that astrocytes have become a central focus in NP research, with the field rapidly evolving from descriptive pathological observations toward mechanism‐based and translational investigations. Future studies should place greater emphasis on disease‐specific astrocytic phenotypes, pathological mechanisms, and clinically translatable therapeutic strategies. Integrating bibliometric insights with mechanistic neuroscience may help accelerate the development of targeted interventions for chronic NP.

**Nomenclature**
AGE: Advanced glycation end product
CCI: Chronic constriction injury
CIPN: Chemotherapy‐induced peripheral neuropathy
EAAT2: Excitatory amino acid transporter 2
GFAP: Glial fibrillary acidic protein
GLT‐1: Glutamate transporter 1
IL‐1β: Interleukin‐1 beta
MAPK: Mitogen‐activated protein kinase
MeSH: Medical Subject Headings
NC: Number of citations
NDRG2: N‐Myc downstream regulated gene 2
NoP: Number of publications
NP: Neuropathic pain
SCI: Spinal cord injury
SCP: Single‐country publications
MCP: Multiple‐country publications
SNI: Spared nerve injury
TC: Total citations
TNF‐α: Tumor necrosis factor‐alpha
WoSCC: Web of Science Core Collection

## Author Contributions

Li Xue: data curation; formal analysis; methodology; software; writing–original draft; and writing–reviewing and editing; Haixia Fan: writing–reviewing, and editing; Hong Yuan: software; supervision; and visualization; Jiao Yang: software; supervision; and visualization; and Qiurui Yuan: software; supervision; and visualization.

## Funding

No funding was obtained for this manuscript.

## Disclosure

This manuscript has previously been posted as a preprint on medRxiv [40] at https://www.medrxiv.org/content/10.64898/2026.01.23.26344689v1.

## Conflicts of Interest

The authors declare no conflicts of interest.

## Supporting Information

Additional supporting information can be found online in the Supporting Information section.

## Supporting information


**Supporting Information** Supporting Table 1 presents a comprehensive bibliometric overview of studies on astrocytes in neuropathic pain published from 2000 to 2025, covering key metrics including publication timeline, document sources, output volume, citation patterns, author contributions, and document types. Supporting Table 2 lists the top 25 countries/regions contributing to research on astrocytes in neuropathic pain, sorted by corresponding author output, with detailed data on publication counts, percentage shares, and single‐country/multicountry publication (SCP/MCP) ratios. Supporting Table 3 details the data search strategies used in the WoSCC and Scopus databases.

## Data Availability

The data supporting the findings of this study are available upon request from the corresponding author.
