# Supplementary material for: Astrocytes in Neuropathic Pain: Mechanistic and Global Insights
Source: Pain Res Manag. 2026 Sep 18;2026:7987792. doi: 10.1155/prm/7987792 (PMC13589199; doi:10.1155/prm/7987792)
Supplement: Supplementary file 1 [file PRM-2026-7987792-s001.docx]

**Supplementary Table 1.** Bibliometric Overview of Astrocytes in Neuropathic Pain (2000–2025)

**Supplementary Table 2.** Most Relevant Countries by Corresponding Author Contributions (2000–2025)

**Supplementary Table 3.** Data search expressions

**Supplementary Table 1**. Bibliometric Overview of Astrocytes in Neuropathic Pain (2000–2025)

| **Description** | **Results** |
| --- | --- |
| MAIN INFORMATION ABOUT DATA |  |
| Timespan | 2000:2025 |
| Sources (Journals, Books, etc) | 483 |
| Documents | 1828 |
| Annual Growth Rate % | 15.24 |
| Document Average Age | 7.23 |
| Average citations per doc | 55.86 |
| References | 31801 |
| DOCUMENT CONTENTS |  |
| Keywords Plus (ID) | 11601 |
| Author's Keywords (DE) | 3576 |
| AUTHORS |  |
| Authors | 6687 |
| Authors of single-authored docs | 48 |
| AUTHORS COLLABORATION |  |
| Single-authored docs | 52 |
| Co-Authors per Doc | 6.5 |
| International co-authorships % | 4.431 |
| DOCUMENT TYPES |  |
| article | 1774 |
| review | 54 |

**Supplementary Table 2.** Most Relevant Countries by Corresponding Author Contributions

| Rank | **Country** | **Articles** | **Articles %** | **SCP** | **MCP** | **MCP %** |
| --- | --- | --- | --- | --- | --- | --- |
| 1 | CHINA | 239 | 13.1 | 227 | 12 | 5 |
| 2 | USA | 94 | 5.1 | 74 | 20 | 21.3 |
| 3 | KOREA | 88 | 4.8 | 75 | 13 | 14.8 |
| 4 | JAPAN | 63 | 3.4 | 53 | 10 | 15.9 |
| 5 | ITALY | 26 | 1.4 | 23 | 3 | 11.5 |
| 6 | AUSTRALIA | 15 | 0.8 | 11 | 4 | 26.7 |
| 7 | POLAND | 13 | 0.7 | 13 | 0 | 0 |
| 8 | BRAZIL | 9 | 0.5 | 7 | 2 | 22.2 |
| 9 | CANADA | 9 | 0.5 | 8 | 1 | 11.1 |
| 10 | IRAN | 9 | 0.5 | 8 | 1 | 11.1 |
| 11 | FRANCE | 8 | 0.4 | 7 | 1 | 12.5 |
| 12 | RUSSIA | 8 | 0.4 | 8 | 0 | 0 |
| 13 | UNITED KINGDOM | 8 | 0.4 | 5 | 3 | 37.5 |
| 14 | GERMANY | 5 | 0.3 | 5 | 0 | 0 |
| 15 | NETHERLANDS | 5 | 0.3 | 2 | 3 | 60 |
| 16 | SPAIN | 5 | 0.3 | 3 | 2 | 40 |
| 17 | SWEDEN | 3 | 0.2 | 3 | 0 | 0 |
| 18 | CZECH REPUBLIC | 2 | 0.1 | 1 | 1 | 50 |
| 19 | DENMARK | 2 | 0.1 | 1 | 1 | 50 |
| 20 | FINLAND | 2 | 0.1 | 1 | 1 | 50 |
| 21 | HUNGARY | 2 | 0.1 | 1 | 1 | 50 |
| 22 | KUWAIT | 2 | 0.1 | 2 | 0 | 0 |
| 23 | SWITZERLAND | 2 | 0.1 | 2 | 0 | 0 |
| 24 | BULGARIA | 1 | 0.1 | 1 | 0 | 0 |
| 25 | CHILE | 1 | 0.1 | 1 | 0 | 0 |

**Supplementary Table 3.** Data search expressions

| **Database** | **Expression** |
| --- | --- |
| WoSCC | （TS="Neuralgias" OR "Neurodynia" OR "Neurodynias" OR "Neuropathic Pain" OR "Neuropathic Pains" OR "Pain, Neuropathic" OR "Pains, Neuropathic" OR "Nerve Pain" OR "Nerve Pains" OR "Pain, Nerve" OR "Pains, Nerve" OR "Paroxysmal Nerve Pain" OR "Nerve Pain, Paroxysmal" OR "Nerve Pains, Paroxysmal" OR "Pain, Paroxysmal Nerve" OR "Pains, Paroxysmal Nerve" OR "Paroxysmal Nerve Pains" OR "Neuralgia, Atypical" OR "Atypical Neuralgia" OR "Atypical Neuralgias" OR "Neuralgias, Atypical" OR "Neuralgia, Iliohypogastric Nerve" OR "Iliohypogastric Nerve Neuralgia" OR "Iliohypogastric Nerve Neuralgias" OR "Nerve Neuralgia, Iliohypogastric" OR "Nerve Neuralgias, Iliohypogastric" OR "Neuralgias, Iliohypogastric Nerve" OR "Neuralgia, Ilioinguinal" OR "Ilioinguinal Neuralgia" OR "Ilioinguinal Neuralgias" OR "Neuralgias, Ilioinguinal" OR "Neuralgia, Perineal" OR "Neuralgias, Perineal" OR "Perineal Neuralgia" OR "Perineal Neuralgias" OR "Neuralgia, Stump" OR "Neuralgias, Stump" OR "Stump Neuralgia" OR "Stump Neuralgias" OR "Neuralgia, Supraorbital" OR "Neuralgias, Supraorbital" OR "Supraorbital Neuralgia" OR "Supraorbital Neuralgias" OR "Neuralgia, Vidian" OR "Neuralgias, Vidian" OR "Vidian Neuralgia" OR "Vidian Neuralgias"）AND（TS="Astrocyte" OR "Astroglia Cells" OR "Astroglia Cell" OR "Cell, Astroglia" OR "Astroglial Cells" OR "Astroglial Cell" OR "Cell, Astroglial" OR "Astroglia" OR "Astroglias"） |
| Scopus | （TI=（"Neuralgias" OR "Neurodynia" OR "Neurodynias" OR "Neuropathic Pain" OR "Neuropathic Pains" OR "Pain, Neuropathic" OR "Pains, Neuropathic" OR "Nerve Pain" OR "Nerve Pains" OR "Pain, Nerve" OR "Pains, Nerve" OR "Paroxysmal Nerve Pain" OR "Nerve Pain, Paroxysmal" OR "Nerve Pains, Paroxysmal" OR "Pain, Paroxysmal Nerve" OR "Pains, Paroxysmal Nerve" OR "Paroxysmal Nerve Pains" OR "Neuralgia, Atypical" OR "Atypical Neuralgia" OR "Atypical Neuralgias" OR "Neuralgias, Atypical" OR "Neuralgia, Iliohypogastric Nerve" OR "Iliohypogastric Nerve Neuralgia" OR "Iliohypogastric Nerve Neuralgias" OR "Nerve Neuralgia, Iliohypogastric" OR "Nerve Neuralgias, Iliohypogastric" OR "Neuralgias, Iliohypogastric Nerve" OR "Neuralgia, Ilioinguinal" OR "Ilioinguinal Neuralgia" OR "Ilioinguinal Neuralgias" OR "Neuralgias, Ilioinguinal" OR "Neuralgia, Perineal" OR "Neuralgias, Perineal" OR "Perineal Neuralgia" OR "Perineal Neuralgias" OR "Neuralgia, Stump" OR "Neuralgias, Stump" OR "Stump Neuralgia" OR "Stump Neuralgias" OR "Neuralgia, Supraorbital" OR "Neuralgias, Supraorbital" OR "Supraorbital Neuralgia" OR "Supraorbital Neuralgias" OR "Neuralgia, Vidian" OR "Neuralgias, Vidian" OR "Vidian Neuralgia" OR "Vidian Neuralgias"）OR AB=（"Neuralgias" OR "Neurodynia" OR "Neurodynias" OR "Neuropathic Pain" OR "Neuropathic Pains" OR "Pain, Neuropathic" OR "Pains, Neuropathic" OR "Nerve Pain" OR "Nerve Pains" OR "Pain, Nerve" OR "Pains, Nerve" OR "Paroxysmal Nerve Pain" OR "Nerve Pain, Paroxysmal" OR "Nerve Pains, Paroxysmal" OR "Pain, Paroxysmal Nerve" OR "Pains, Paroxysmal Nerve" OR "Paroxysmal Nerve Pains" OR "Neuralgia, Atypical" OR "Atypical Neuralgia" OR "Atypical Neuralgias" OR "Neuralgias, Atypical" OR "Neuralgia, Iliohypogastric Nerve" OR "Iliohypogastric Nerve Neuralgia" OR "Iliohypogastric Nerve Neuralgias" OR "Nerve Neuralgia, Iliohypogastric" OR "Nerve Neuralgias, Iliohypogastric" OR "Neuralgias, Iliohypogastric Nerve" OR "Neuralgia, Ilioinguinal" OR "Ilioinguinal Neuralgia" OR "Ilioinguinal Neuralgias" OR "Neuralgias, Ilioinguinal" OR "Neuralgia, Perineal" OR "Neuralgias, Perineal" OR "Perineal Neuralgia" OR "Perineal Neuralgias" OR "Neuralgia, Stump" OR "Neuralgias, Stump" OR "Stump Neuralgia" OR "Stump Neuralgias" OR "Neuralgia, Supraorbital" OR "Neuralgias, Supraorbital" OR "Supraorbital Neuralgia" OR "Supraorbital Neuralgias" OR "Neuralgia, Vidian" OR "Neuralgias, Vidian" OR "Vidian Neuralgia" OR "Vidian Neuralgias"）OR AK=（"Neuralgias" OR "Neurodynia" OR "Neurodynias" OR "Neuropathic Pain" OR "Neuropathic Pains" OR "Pain, Neuropathic" OR "Pains, Neuropathic" OR "Nerve Pain" OR "Nerve Pains" OR "Pain, Nerve" OR "Pains, Nerve" OR "Paroxysmal Nerve Pain" OR "Nerve Pain, Paroxysmal" OR "Nerve Pains, Paroxysmal" OR "Pain, Paroxysmal Nerve" OR "Pains, Paroxysmal Nerve" OR "Paroxysmal Nerve Pains" OR "Neuralgia, Atypical" OR "Atypical Neuralgia" OR "Atypical Neuralgias" OR "Neuralgias, Atypical" OR "Neuralgia, Iliohypogastric Nerve" OR "Iliohypogastric Nerve Neuralgia" OR "Iliohypogastric Nerve Neuralgias" OR "Nerve Neuralgia, Iliohypogastric" OR "Nerve Neuralgias, Iliohypogastric" OR "Neuralgias, Iliohypogastric Nerve" OR "Neuralgia, Ilioinguinal" OR "Ilioinguinal Neuralgia" OR "Ilioinguinal Neuralgias" OR "Neuralgias, Ilioinguinal" OR "Neuralgia, Perineal" OR "Neuralgias, Perineal" OR "Perineal Neuralgia" OR "Perineal Neuralgias" OR "Neuralgia, Stump" OR "Neuralgias, Stump" OR "Stump Neuralgia" OR "Stump Neuralgias" OR "Neuralgia, Supraorbital" OR "Neuralgias, Supraorbital" OR "Supraorbital Neuralgia" OR "Supraorbital Neuralgias" OR "Neuralgia, Vidian" OR "Neuralgias, Vidian" OR "Vidian Neuralgia" OR "Vidian Neuralgias"））AND （TI=（"Astrocyte" OR "Astroglia Cells" OR "Astroglia Cell" OR "Cell, Astroglia" OR "Astroglial Cells" OR "Astroglial Cell" OR "Cell, Astroglial" OR "Astroglia" OR "Astroglias"） OR AB=（"Astrocyte" OR "Astroglia Cells" OR "Astroglia Cell" OR "Cell, Astroglia" OR "Astroglial Cells" OR "Astroglial Cell" OR "Cell, Astroglial" OR "Astroglia" OR "Astroglias"） OR AK=（"Astrocyte" OR "Astroglia Cells" OR "Astroglia Cell" OR "Cell, Astroglia" OR "Astroglial Cells" OR "Astroglial Cell" OR "Cell, Astroglial" OR "Astroglia" OR "Astroglias"）） |
